# Integration of chemotherapy into current treatment strategies for brain metastases from solid tumors

**DOI:** 10.1186/1748-717X-1-19

**Published:** 2006-06-27

**Authors:** Carsten Nieder, Anca L Grosu, Sabrina Astner, Reinhard Thamm, Michael Molls

**Affiliations:** 1 Department of Radiation Oncology, Klinikum rechts der Isar der Technischen Universität München, Ismaninger Str. 22, 81675 Munich, Germany

## Abstract

Patients with brain metastases represent a heterogeneous group where selection of the most appropriate treatment depends on many patient- and disease-related factors. Eventually, a considerable proportion of patients are treated with palliative approaches such as whole-brain radiotherapy. Whole-brain radiotherapy in combination with chemotherapy has recently gained increasing attention and is hoped to augment the palliative effect of whole-brain radiotherapy alone and to extend survival in certain subsets of patients with controlled extracranial disease and good performance status. The randomized trials of whole-brain radiotherapy vs. whole-brain radiotherapy plus chemotherapy suggest that this concept deserves further study, although they failed to improve survival. However, survival might not be the most relevant endpoint in a condition, where most patients die from extracranial progression. Sometimes, the question arises whether patients with newly detected brain metastases and the indication for systemic treatment of extracranial disease can undergo standard systemic chemotherapy with the option of deferred rather than immediate radiotherapy to the brain. The literature contains numerous small reports on this issue, mainly in malignant melanoma, breast cancer, lung cancer and ovarian cancer, but very few sufficiently powered randomized trials. With chemotherapy alone, response rates were mostly in the order of 20–40%. The choice of chemotherapy regimen is often complicated by previous systemic treatment and takes into account the activity of the drugs in extracranial metastatic disease. Because the blood-brain barrier is partially disrupted in most macroscopic metastases, systemically administered agents can gain access to such tumor sites. Our systematic literature review suggests that both chemotherapy and radiochemotherapy for newly diagnosed brain metastases need further critical evaluation before standard clinical implementation. A potential chemotherapy indication might exist as palliative option for patients who have progressive disease after radiotherapy.

## Background

**Figure 1:**
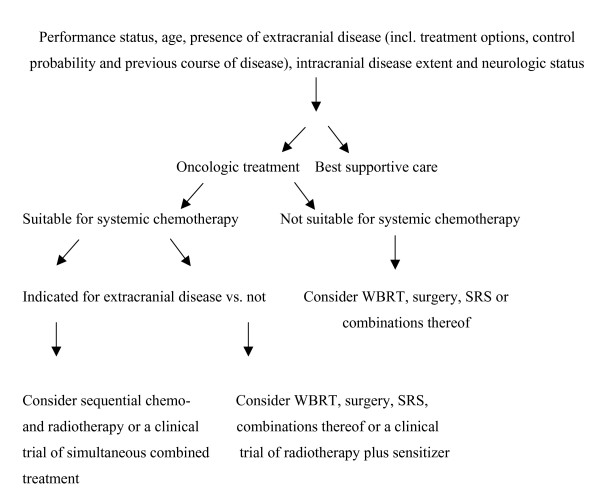
Overview of factors influencing treatment decisions in patients with newly diagnosed brain metastases. The algorithm is based on results of published clinical trials with various levels of evidence (not all questions have been addressed in randomized controlled trials so far) and reflects the current practice in the authors' institution.

Local control of a limited number (mostly 1–3, in some series >3) of brain metastases can effectively be achieved by surgical resection or stereotactic radiosurgery (SRS) with or without adjuvant whole-brain radiotheray (WBRT) [1-9] (Table 1). The number of patients dying from uncontrolled brain metastases despite such intensive local treatment is comparably low and ranges from 20–30%. However, patients with brain metastases are a heterogeneous group where selection of the most appropriate treatment depends on many patient- and disease-related factors. Figure 1 provides an overview of potential factors influencing decision making. Eventually, a considerable proportion of patients with multiple brain metastases, which are not suitable for surgery or SRS, might be candidates for other palliative approaches such as WBRT alone or combined with chemotherapy. The latter combination has recently gained increasing attention and is hoped to augment the palliative effect of WBRT alone and to extend survival in certain subsets of patients. Certainly, maximing local control within the brain is most important in case of controlled extracranial disease and good performance status. So far, data from controlled clinical trials of combined chemo- and radiotherapy are still limited. The choice of chemotherapy regimen is often complicated by previous systemic treatment and takes into account the activity of the drugs in extracranial metastatic disease and the issue of drug concentration within the central nervous system, although it has been realized that the blood-brain barrier (BBB) is partially disrupted in most macroscopic metastases. Thus, systemically administered agents can gain access to such tumor sites. Sometimes, the question arises whether patients with newly detected brain metastases and the indication for systemic treatment of extracranial disease can undergo standard systemic chemotherapy with the option of deferred rather than immediate radiotherapy to the brain. The literature contains numerous small reports on this issue, mainly in malignant melanoma, breast cancer, lung cancer and ovarian cancer, but very few sufficiently powered randomized trials [10,11]. In order to give treatment recommendations, we have systematically reviewed the results of both chemotherapy alone and combined with radiation treatment for newly diagnosed brain metastases from solid tumors except germ cell malignancies.

**Table 1:** Results of surgery and stereotactic radiosurgery (SRS) for brain metastases

Reference	n (patients and lesions)	Prescribed dose (median; range [Gy])*	Median OS	1-year PFS (%)
Patchell et al. 1990 [1]	25/25	Surgery	9.5	80
Patchell et al. 1998 [2]	49/49	Surgery	11.0	82
Pirzkall et al. 1998 [3]	236/311	20; 10–30	5.5	89
Cho et al. 1998 [4]	73/136	17.5; 6–50	7.8	80
Kocher et al. 1998 [5]	106/157	20; 12–25	8.0	85
Sneed et al. 1999 [6]	62/118^a^ 43/117^b^	18; 15–22 17.5; 15–22	11.3 11.1	80 86
Varlotto et al. 2003 [7]	137/208	16; 12–25	Not given	90
Andrews et al. 2004 [8]	164/269^c^	Not given; 15–24	6.5	82
Bhatnagar et al. 2006 [9]	205/4-18 lesions each^d^	16; 12–20	8.0	71

OS: overall survival in months; PFS: progression-free survival; ?: data not reported

* Prescription isodose or point varied, some series included SRS plus WBRT

^a ^SRS only

^b ^SRS plus WBRT (no significant difference in OS and PFS between both groups)

^c ^SRS plus WBRT

^d ^SRS plus/minus WBRT

## Methods

This review compares the results of clinical trials of chemotherapy or combined radio- and chemotherapy for brain metastases, based on a systematic literature search by use of Medline (Pub Med by the National Library of Medicine, National Institutes of Health, Bethesda, Maryland, USA, last access March 31, 2006). Studies were identified by entering combinations of the keywords "radiotherapy or chemotherapy" and "brain metastases or cerebral metastases". In addition, the reference lists of all articles and the abstracts of the annual meeting 2005 of the American Society of Clinical Oncology and the American Society for Therapeutic Radiology and Oncology were searched. From all published studies, prespecified variables were extracted and compared.

## Results

Agents investigated so far include cisplatin and cisplatin combinations (with teniposide, etoposide, taxanes, or vinorelbine), paclitaxel, topotecan, temozolomide, nitrosoureas and various combinations of these. With chemotherapy alone, response rates were mostly in the order of 20–40% (Table 2[10,12-25]). Taking into account the non-randomized design of these trials and the limited patient numbers, none of these regimens is clearly superior to the others. Most studies reporting on this issue found comparable response rates in extracranial disease sites if patients had both intra- and extracranial disease. Thus, the choice of treatment can be guided by individual factors such as previous regimens, presence of extracranial disease and response rates in extracranial disease and tolerance/organ function. Even in responding patients with brain metastases, the effect of chemotherapy was transient and often limited to 3–6 months. Median survival was 3–10 months. The difference between median time to progression or progression-free survival on the one hand and median overall survival on the other hand was variable, ranging from 0.5 to 4.6 months in the 10 studies that reported on these endpoints (median 2.65 months). Thus, it is very likely that additional treatment was given after progression in many studies. However, information about such treatment is not available in the articles. No systematic evaluation of neurotoxicity or quality of life after chemotherapy is available yet.

**Table 2:** Results of chemotherapy for brain metastases (some trials also included patients with previous radiotherapy)

Reference	n (patients)	Regimen	OR rate	Median TTP	Median OS
Bafaloukos et al. 2004 [12]	25 melanoma	Temozolomide alone or plus cisplatin or docetaxel	24%	2.0	4.7
Hwu et al. 2005 [13]	26 melanoma	Temozolomide plus thalidomide	12%	Not given	5.0
Agarwala et al. 2004 [14]	151 melanoma	Temozolomide alone	7%	1.1 (PFS)	3.2
Christodoulou et al. 2001 [15]	28 various	Temozolomide alone	4%	3.0	4.5
Abrey et al. 2001 [16]	41 various	Temozolomide alone	6%	2.0	6.6
Caraglia et al. 2006 [17]	19 various	Temozolomide plus pegylated liposomal doxorubicin	37%	5.5 (PFS)	10.0
Christodoulou et al. 2005 [18]	32 various	Temozolomide plus cisplatin	31%	2.9	5.5
Oberhoff et al. 2001 [19]	24 breast ca	Topotecan	25%	4.1 (response duration)	6.3
Korfel et al. 2002 [20]	30 SCLC	Topotecan	33%	3.1	3.6
Bernardo et al. 2002 [21]	22 NSCLC	Vinorelbine plus gemcitabine and carboplatin	45%	5.7 (response duration)	7.6
Cortes et al. 2003 [10]	26 NSCLC	Paclitaxel/cisplatin plus either vinorelbine or gemcitabine	38%	2.9	4.9*
Franciosi et al. 1999 [22]	116 various	Cisplatin plus etoposide	38%^1^ 30%^2^ 0%^3^	3.9 3.9 2.5	7.1 7.3 3.9
Jacquillat et al. 1990 [23]	36 melanoma	Fotemustine	25%	Not given	Not given
Boogerd et al. 1992 [24]	22 breast ca	Cyclophosphamide, 5-fluoro-uracil and methotrexate or doxorubicin	55%	Not given	5.7
Kaba et al. 1997 [25]	97 various	Thioguanine, procarbazine, dibromodulcitol, CCNU, fluorouracil and hydroxyurea	28%	2.8	5.7

OR: objective response; OS: overall survival in months; TTP: time to progression in months; PFS: progression-free survival in months; SCLC: small cell lung cancer

^1 ^Breast cancer

^2 ^Non-small cell lung cancer (NSCLC)

^3 ^Melanoma

* 15/26 patients had received whole-brain radiotherapy with 30 Gy, 5 additional radiosurgery after chemotherapy

The following clinical trials deserve further discussion because their design included randomization. A study in brain metastases from non-small cell lung cancer (NSCLC) compared these strategies: arm A (n = 86) received cisplatin 100 mg/m^2 ^on day 1 plus vinorelbine 30 mg/m^2 ^on day 1, 8, 15 and 22 (repeated every 4 weeks) [11]. After 2 cycles, responders continued with up to 4 additional cycles. Non-responders received WBRT with 10 fractions of 3 Gy. In Arm B (n = 85), simultaneous WBRT with 30 Gy started on day 1 of the first chemotherapy cycle. There was no significant difference between simultaneous and deferred WBRT in terms of response of brain metastases (27 vs. 33%) and median overall survival (24 vs. 21 weeks). Another randomized study with 120 patients with brain metastases from small-cell lung cancer (SCLC) compared teniposide 120 mg/m^2 ^3× per week every 3 weeks to the same chemotherapy plus WBRT with 10 fractions of 3 Gy [26]. WBRT started within 3 weeks of the first teniposide administration. In this study, the response rate (22 vs. 57%) and time to progression of brain metastases were significantly worse after chemotherapy alone, however, survival was comparable. Mornex et al. randomized 76 patients with brain metastases from malignant melanoma to either fotemustine or fotemustine plus concomitant WBRT with 15 fractions of 2.5 Gy [27]. There was a significant difference in favour of combined treatment for the time to cerebral progression and a trend for both control rates at 7 weeks (30% vs. 47%) and overall survival, which was 22% longer after combined treatment. Response rates were equally low in both arms (7.4% vs. 10%).

A small randomized study with 52 patients evaluated WBRT with 20 fractions of 2 Gy vs. combined WBRT and temozolomide 75 mg/m^2^/day [28]. In the combined modality arm, temozolomide continued for 6 more cycles (200 mg/m^2^/day for 5 days every 4 weeks). There was a significantly higher response rate in the temozolomide arm resulting from an increased number of partial remissions (96 vs. 67%). The influence on overall survival was not significant (7 vs. 8.6 months). A second randomized trial of temozolomide (75 mg/m^2^/day and two additional cycles with 200 mg/m^2^/day for 5 days every 4 weeks) plus WBRT (30 Gy) was designed as a phase II study with 82 patients and therefore also does not allow to draw definitive conclusions [29]. Overall survival and response rates were similar, while progression-free survival at 90 days was better for combined treatment (72 vs. 54%, p = 0.03). Death from brain metastases was more common after WBRT alone (69 vs. 41%, p = 0.03). An older randomized trial from Japan compared WBRT alone to WBRT plus nitrosoureas and WBRT plus nitrosoureas and tegafur in 100 patients with lung cancer [30]. The trial also included patients treated after surgical resection. The objective response rate was significantly improved (more than doubled) when WBRT alone was compared to WBRT plus nitrosourea and tegafur. In all 3 groups, most patients died from systemic disease progression and no significant difference in survival was found. Chemotherapy with low-dose WBRT does not seem to be an attractive option, as illustrated in a randomized trial that was closed prematurely after 42 patients with NSCLC because of poor accrual [31]. In that study, daily carboplatin was added to WBRT with 5 fractions of 4 Gy. Median OS was 4.4 vs. 3.7 months with disappointing response rates of 10 vs. 29%. Topotecan daily i.v. in addition to WBRT has been evaluated in a phase I/II trial [32]. Median OS was 5 months, CR+PR rate in assessable patients 58%. This drug is currently under further investigation. In 40 patients with melanoma metastases, WBRT with 10 fractions of 3 Gy plus temozolomide and thalidomide produced relatively disappointing results [33]. CR+PR rate was 3%, median time to progression 10 weeks and median survival 4 months.

Other approaches for radiosensitization of tumor cells in conjunction with WBRT investigated the drugs efaproxiral, which modifies tumor oxygenation [34], motexafin gadolinium [35], a paramagnetic redox active drug, and celecoxib [36], a cyclooxygenase-2 inhibitor. In a large randomized phase III study, efaproxiral significantly improved the survival of the patient subgroup with breast cancer [34]. Therefore, a confirmatory trial in this population has been initiated. With motexafin gadolinium, the subgroup with non-small cell lung cancer had significantly longer time to neurologic progression [35]. A confirmatory randomized phase III trial has been completed and awaits publication. Celecoxib was given concomitant to accelerated-hyperfractionated WBRT plus boost in a phase I/II study with 27 patients [36]. The results are promising (complete plus partial responses 67%, median time to neurological progression 6 months, median survival 8.7 months). Whether this results from patient selection, radiotherapy to more than 54 Gy, or the drug needs clarification in additional trials.

## Discussion

Systemic chemotherapy with different agents has been studied in often relatively small and heterogeneous groups of patients. It was found to induce objective remissions in a minority of these patients and it appears that WBRT or WBRT plus chemotherapy results in higher response rates [26,28,32,35-39], although such comparison might be subject to selection bias and needs confirmation in prospective randomized trials. Even if systemic chemotherapy is indicated for advanced extracranial lesions, WBRT can be administered between two cycles. In case of progression after WBRT, systemic chemotherapy might offer palliation, as described by Abrey et al. who treated 41 patients with temozolomide [16]. Twenty of these patients also had surgery or radiosurgery in addition to WBRT and only 6 had no prior chemotherapy (Table 2). In other series, smaller groups of patients with previous WBRT were included [23,24]. Again, occasional responses were seen.

While chemotherapy alone might not be the preferable option in first-line treatment, simultaneously administered agents can be used to enhance the effect of radiotherapy aiming either at additive cell kill or true radiosensitization. The main prerequisites of successful chemotherapy are sensitivity of the tumor cells to the mechansims of the drug and sufficient drug exposure. The key issues of tumor heterogeneity with primary and acquired resistance as well as pharmacokinetics, pharmacodynamics and tumor microenvironment deserve particular attention because of several facts that are specific for brain tumors [40]. First of all, the intact BBB prevents access to the brain for several compounds. Even in areas of BBB disturbance, the effects of contemporary drug treatment are not fully satisfactory. Thus, achieving therapeutic concentrations in distal, seemingly intact areas that also are known to contain tumor cells remains an enormous challenge. Various strategies of modified application or increased dose have been explored, including intraarterial, intrathecal and intratumoral delivery as well as disruption of the BBB. Regarding patients with brain metastases, no definitve recommendations for any of these strategies can be given. Importantly, some patients with brain metastases are able to metabolize certain chemotherapy drugs more rapidly than other tumor patients because of concomitant enzyme-inducing medications that are necessary to treat or prevent seizures. Phenytoin, carbamazepine and phenobarbital induce hepatic cytochrome P450 enzymes, resulting for example in higher maximum tolerated drug doses.

The randomized trials of WBRT vs. WBRT plus chemotherapy by Antonadou et al. [28], Verger et al. [29] and Ushio et al. [30] suggest that this concept deserves further study, although they failed to improve survival. However, survival might not be the most relevant endpoint in a condition, where most patients die from extracranial progression. It is also important to administer a WBRT schedule that kills a large proportion of tumor cells, which is not the case for 10 fractions of 3 Gy or equivalent hypofractionated regimens. When designing new trials to proof the concept of simultaneous radiochemotherapy for brain metastases, the following key questions need to be adressed: what are the most relevant study endpoints, what is the price in terms of toxicity, quality of life and cost, what are the most relevant WBRT and drug administration regimens?

## Conclusion

Three randomized trials of WBRT vs. WBRT plus chemotherapy failed to improve survival. However, neurologic progression-free survival or quality of life might be more relevant endpoints, because most patients die from extracranial progression. Further prospective data on these endpoints are needed. The literature contains numerous small reports but few sufficiently powered randomized trials of systemic chemotherapy with deferred rather than immediate radiotherapy to the brain. With chemotherapy alone, response rates were mostly in the order of 20–40%. Even in responding patients, the effect was transient and often limited to 3–6 months. Median survival was 3–10 months. The choice of chemotherapy regimen is often complicated by previous systemic treatment and takes into account the activity of the drugs in extracranial metastatic disease. New radiosensitizers such as efaproxiral, motexafin gadolinium and celecoxib administered simultaneously to WBRT are currently under investigation. From todays's point of view, chemotherapy and radiochemotherapy for newly diagnosed brain metastases need to undergo further critical evaluation before standard clinical implementation. To detect a potential benefit, the efficacy of the radiotherapy schedule should not be too low. A potential chemotherapy indication might exist as palliative option for patients who have progressive disease after radiotherapy.

## Competing interests

The author(s) declare that they have no competing interests.

## Authors' contributions

Conception and design: CN, ALG, MM

Data acquisition and analysis: SA, RT

Data interpretation: SA, RT, CN

Manuscript preparation: CN, ALG, MM

All authors read and approved the final manuscript.
